# Individual factors associated with physical activity adherence in schizophrenia: an exploratory secondary analysis of two randomized controlled trials

**DOI:** 10.3389/fpsyg.2026.1878217

**Published:** 2026-07-28

**Authors:** Susanne Kuprat, Lena Deller, Johanna Spaeth, Deniz Yilmaz, Mona Hussain, Camilla Brandt, Lukas Roell, Astrid Roeh, Andrea Schmitt, Peter Falkai, Isabel Maurus, The BrainTrain Working Group, M. Amann, J. Bauerreiß, J. Diegelmann, J. Glad, J. Jannan, M. John, E. B. Kara, Ö. Koc, K. Lada, J. Moussiopoulou, L. Sagstetter, J. Sautner, J. Segerer, N. Theis, J. Wartensleben von, A. Weibel, L. Widmann, V. Yakimov, D. Yildrim, M. Zuliani

**Affiliations:** 1 Department of Psychiatry and Psychotherapy, LMU University Hospital, LMU Medizin, Ludwig-Maximilians-Universität München, Munich, Germany; 2 Max Planck School of Cognition, Max Planck Institute for Human Cognitive and Brain Sciences, Leipzig, Germany; 3 NeuroImaging Core Unit Munich (NICUM), LMU University Hospital, Ludwig-Maximilians-Universität München, Munich, Germany; 4 kbo-Isar-Amper-Klinikum München-Nord, Munich, Germany; 5 Department of Psychiatry, The University of Melbourne, Melbourne, VIC, Australia; 6 Department of Psychiatry, Psychotherapy and Psychosomatics of the University Augsburg, Medical Faculty, Bezirkskrankenhaus Augsburg, University of Augsburg, Augsburg, Germany; 7 Max Planck Institute of Psychiatry, Munich, Germany; 8 German Center for Mental Health (DZPG), Partner site Munich/ Augsburg, Germany; 9 Laboratory of Neurosciences (LIM-27), Institute of Psychiatry, University of São Paulo (USP), São Paulo, Brazil

**Keywords:** exercise adherence, motivation, personality traits, physical activity, schizophrenia

## Abstract

**Introduction:**

Physical activity is an effective adjunctive therapy in schizophrenia, improving psychiatric symptoms and reducing the mortality rate. However, individuals with schizophrenia engage in lower levels of physical activity compared to the general population, and adherence to structured exercise programs remains low. The factors underlying sustained engagement are still not well understood. This study aimed to investigate factors associated with adherence to an exercise intervention in individuals with schizophrenia and to explore whether personality characteristics are related to physical fitness outcomes.

**Methods:**

The present investigation was a secondary analysis of data from two randomized controlled trials conducted at the LMU Munich, which included *N* = 100 participants. Participants completed a three-month, professionally supervised exercise intervention, consisting of either aerobic endurance training or flexibility, strength, and balance training. Assessments at screening and baseline included sociodemographic characteristics, lactate threshold testing, physical fitness measures, personality and motivational questionnaires, and clinical and functional ratings. Multiple regression analyses were used to examine adherence as the primary outcome and physical fitness as a secondary outcome. The predictor variables included motivation, personality traits, symptom severity, previous exercise experience and emotional support, baseline fitness level, affective forecasting, and satisfaction with exercise training.

**Results:**

The results revealed no significant associations between any of the investigated variables and adherence after correction for multiple testing (all *p* > 0.05). Similarly, physical fitness was not significantly associated with personality- related variables in this study.

**Discussion:**

Overall, the examined variables explained only a limited proportion of the variance in the two outcomes. These findings suggest that the investigated variables do not fully explain adherence or physical fitness and that both outcomes may be influenced by additional contextual determinants of exercise behavior in schizophrenia.

## 1 Introduction

Regular physical activity is an effective non-pharmacological strategy for improving overall health and well-being, with benefits extending beyond somatic conditions to severe mental disorders, such as schizophrenia (Firth et al., 2015; Pedersen and Saltin, 2015). Exercise has been shown to positively affect both physical and mental health outcomes (Dauwan et al., 2016; Maurus et al., 2023), and aerobic training is already recommended in the German clinical guidelines for schizophrenia (Deutsche Gesellschaft für Psychiatrie und Psychotherapie, Psychosomatik und Nervenheilkunde (DGPPN), 2025). Despite these recommendations, individuals with schizophrenia engage in significantly lower levels of physical activity compared to healthy controls (Koepl et al., 2025). Furthermore, adherence to structured exercise interventions remains challenging, with high dropout rates frequently reported in this population (Vancampfort et al., 2016; Schwaiger et al., 2024).

A key challenge is that the factors determining sustained participation in exercise interventions among individuals with schizophrenia are not well understood. Given the complexity of this disorder, which involves impairments across perceptual, cognitive, affective, and motor domains (Deutsche Gesellschaft für Psychiatrie und Psychotherapie, Psychosomatik und Nervenheilkunde (DGPPN), 2025), identifying reliable determinants of exercise adherence remains difficult. Although previous literature has examined a range of potential predictors, evidence of their association with exercise adherence is limited and inconsistent.

One domain that has received particular attention is motivational factors. Among these, autonomous motivation has been identified as an important correlate of physical activity in individuals with schizophrenia and may play a crucial role in daily movements. Self-Determination Theory (SDT) proposes three basic psychological needs, namely autonomy, competence, and relatedness. It also distinguishes between intrinsic and extrinsic motivation (Ryan and Deci, 2017). Within this framework, autonomous motivation refers to behavior driven by internalized values and personal choice rather than external pressure. It has been shown to be more effective in initiating and sustaining long-term behavioral changes (Costa et al., 2018). Accordingly, autonomous motivation has been identified as one of the strongest predictors of weekly physical activity (Vancampfort et al., 2013). In addition, self-efficacy appears to be an important determinant, with higher levels associated with greater activity engagement in individuals with schizophrenia (Vancampfort et al., 2012). Moreover, exercise-related self-efficacy expectations have emerged as strong predictors of physical activity in patients with mental disorders (Koepl et al., 2025).

Beyond motivational factors, personality traits have also been associated with physical activity behavior. In healthy individuals, extraversion and conscientiousness are positively related to physical activity, whereas neuroticism and openness showed mixed or negative associations (Rhodes and Smith, 2006; Sutin et al., 2016). Agreeableness, on the other hand, yielded inconsistent findings with partial effects on initiation and maintenance of physical activity (Caille et al., 2024). In addition to psychological characteristics, prior exercise experience may also influence physical activity engagement. Indeed, childhood and adolescent physical activity levels have been identified as predictors of regular exercise among adults with mental health conditions (Wambsganz et al., 2024). However, individuals with mental illness tend to show lower activity levels during developmental stages (Koepl et al., 2025), suggesting potential long-term behavioral trajectories that may influence intervention engagement.

In schizophrenia, clinical characteristics represent an important source of variability in exercise behavior. Symptom severity, particularly negative symptoms, is negatively associated with physical activity levels (Walther et al., 2009). Furthermore, individuals with higher functioning and lower symptom burden tend to show better adherence to exercise interventions than those with lower functioning and higher symptom severity (Schwaiger et al., 2024). Crucially, the relationship between physical activity and clinical status is bidirectional, with exercise interventions ameliorating psychiatric symptoms (Curcic et al., 2017; Maurus et al., 2023), and psychiatric status in turn influencing engagement in physical activity.

Despite growing evidence on motivational, personality-related, developmental, and clinical factors associated with physical activity behavior, the findings are mixed and it remains unclear which factors are most strongly associated with adherence to structured exercise interventions in individuals with schizophrenia. This knowledge gap is clinically relevant because adherence is a prerequisite for achieving the physical and mental health benefits of exercise interventions. The present secondary analysis addresses this gap by combining data from two exercise intervention studies, BrainTrain (BT) and OligoTreat (OT), involving individuals with schizophrenia spectrum disorders. Pooling these datasets increases the sample size and enables a more comprehensive examination of motivational, personality-related, developmental, and clinical factors associated with exercise adherence.

Accordingly, the present study aimed to identify factors associated with adherence to structured exercise interventions among individuals with schizophrenia spectrum disorders. Furthermore, it sought to generate knowledge that may support strategies promoting sustained physical activity in daily life.

Based on previous literature, it was hypothesized that high levels of intrinsic motivation, extraversion, agreeableness, and conscientiousness would be associated with greater exercise adherence. Additionally, higher baseline fitness levels, prior exercise experience in childhood and adolescence, and greater perceived social support were expected to predict higher adherence, whereas higher symptom severity was expected to be negatively associated with adherence.

In addition to these hypothesis-driven analyses, we explored whether functioning in daily life, affective forecasting and satisfaction with the intervention were associated with exercise adherence and whether personality traits were related to physical fitness outcomes. By identifying factors associated with exercise adherence, this study aimed to contribute to a better understanding of exercise behavior in schizophrenia and to inform future strategies for promoting sustained physical activity.

## 2 Methods

### 2.1 Study design and intervention

The investigation was based on two clinical randomized, controlled clinical trials (RCTs) that evaluated the effects of a 3-months exercise intervention in individuals with schizophrenia, namely the BT and the OT trials.

The BT study examined the effects of physical exercise on clinical symptoms and underlying biological mechanisms in individuals with schizophrenia. The trial was approved by the local ethics committees (project number 22-0921, approval date: May 8, 2023) and is registered in the international clinical trials database (NCT05956327) and the German Clinical Trials Register (DRKS00032187). The study is conducted at the Department of Psychiatry and Psychotherapy of the Ludwig Maximilian University of Munich.

The OT study examined the effects of aerobic exercise combined with clemastine or placebo on clinical and functional outcomes, while additionally targeting biological mechanisms related to myelination. The study is registered in the international clinical trials database (NCT06315972) and the German Register for Clinical Trials (DRKS ID: DRKS00032316). The trial was approved by the local ethics committees (project number 23-0062, approval date: June 2, 2023).

Both studies employed structured aerobic exercise interventions and comparable study frameworks, providing the basis for the pooled analyses conducted in the present study. In the BT study, participants were randomly assigned to either an aerobic endurance exercise group or a flexibility, strength, and balance training group. In the OT study, all participants received the same aerobic endurance training as in the BT study and were randomized to either adjunctive pharmacological treatment (Clemastine) or placebo. In both study programs, endurance training sessions were performed on a bicycle ergometer at a moderate intensity level, individually prescribed based on the results of a lactate threshold test. Warm-up and cool-down phases were performed at 80% of the individually calculated training workload. Moderate intensity was defined as training at the workload corresponding to a blood lactate concentration of 2 mmol/l. All exercise sessions were supervised by experienced exercise professionals and conducted in small groups of up to three participants. The program took place over a three-month period per participant in both studies. Participants could attend a maximum of three sessions per week, for a total of 36 sessions. Depending on the training week, training duration was 40 min (week one and two), 45 min (week three and four) and 50 min (week five to twelve) and included 5 min of warm-up and 5 min of cool-down.

The primary aim of the study was to identify potential associations between exercise adherence and intrinsic motivational aspects as well as personality- structural characteristics in individuals with schizophrenia. Furthermore, childhood and adolescent sports participation and perceived emotional family support were assessed as predictors of adherence to the exercise program. The study also evaluated whether training adherence was influenced by symptom severity, daily life functioning, affective forecasting, and the level of satisfaction with the exercise training. Physical working capacity (PWC) - derived fitness values were treated simultaneously as both, a potential predictor of exercise adherence and an outcome variable in relation to personality traits.

### 2.2 Study participants

The present secondary analysis included a total of *N* = 100 participants recruited from two RCTs: BT (*N* = 71) and OT *(N* = 29). Both studies included male and female patients diagnosed with schizophrenia or schizophrenia spectrum disorders according to DSM-V criteria, as assessed by the Mini-International Neuropsychiatric Interview (M. I. N. I.) (Sheehan et al., 1998). Details of the demographic characteristics are provided in the Results section. Participants were aged between 18 and 65 years. Eligibility criteria included stable antipsychotic treatment, absence of pregnancy or breastfeeding, and the use of adequate contraception in women of childbearing potential. Symptom severity was assessed using the Positive and Negative Syndrome Scale (PANSS) (Kay et al., 1987) and was required not to exceed a score of 75. Detailed inclusion and exclusion criteria are provided in Supplementary Table 1.

### 2.3 Assessments

The screening phase (V0) took place within seven days before randomization and included sociodemographic data collection, the lactate threshold testing, PWC assessment, and personality and motivational questionnaires. To assess motivational aspects, selected subscales of the Exercise Motivation Inventory-2 (EMI-2) (Markland and Ingledew, 1997), including challenge, competition, enjoyment, nimbleness, positive health, revitalization, strength and endurance, and stress management, were used. Internal locus of control was assessed using the Internal-External Control Beliefs Scale- 4 (IE-4 scale), based on the internal control score (Kovaleva et al., 2012). Self-efficacy in relation to sporting activity and movement behavior was assessed using a validated German questionnaire, the Self-efficacy toward physical exercise scale (SSA) (Fuchs and Schwarzer, 1994). The personality traits conscientiousness, agreeableness, extraversion, openness and neuroticism were assessed using the Big Five Inventory (BFI-10) (Rammstedt et al., 2013). In the BT study, a study-specific questionnaire was used to collect retrospective, anamnesis-based information on previous exercise experience, including sports participation in childhood and adolescence as well as perceived emotional support from family members. The lactate threshold test was conducted for physiological characterization and to derive measures of PWC.

At baseline (V1, days 0–7), clinical symptoms were assessed using the PANSS (Kay et al., 1987) and functioning in daily life using the Global Assessment of Functioning (GAF) (Endicott et al., 1976). Additionally, subjective expectations regarding the intervention’s effectiveness were assessed using the Affective Forecasting Questionnaire, developed within the BT trial. The questionnaire covered expectations of improvements in clinical symptoms, cognitive functioning, overall functioning, quality of life, physical health, and brain structure and function.

At V2 (days 30–37), a study-specific questionnaire, originally designed for the BT trial, was administered to assess participants’ satisfaction with the exercise training during the intervention.

**FIGURE 1:**
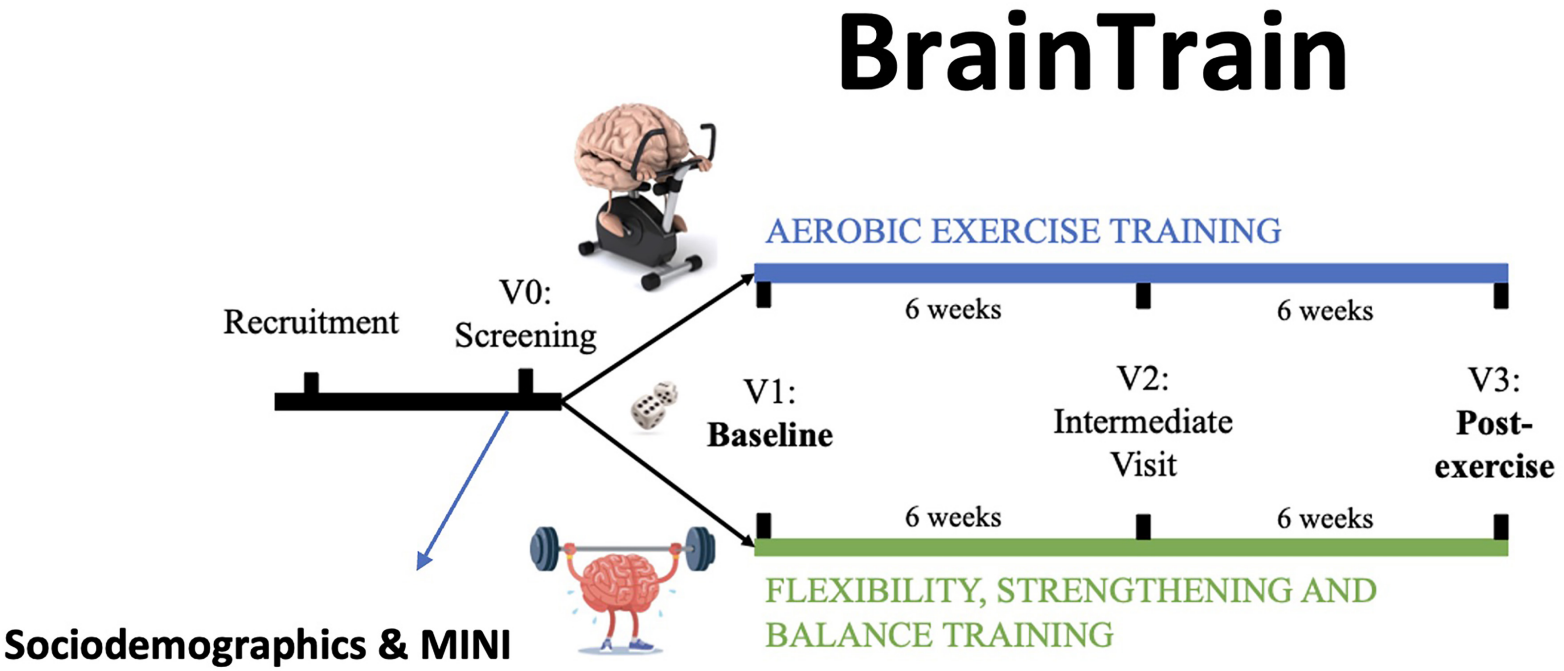
Study timeline of BT.

**FIGURE 2:**
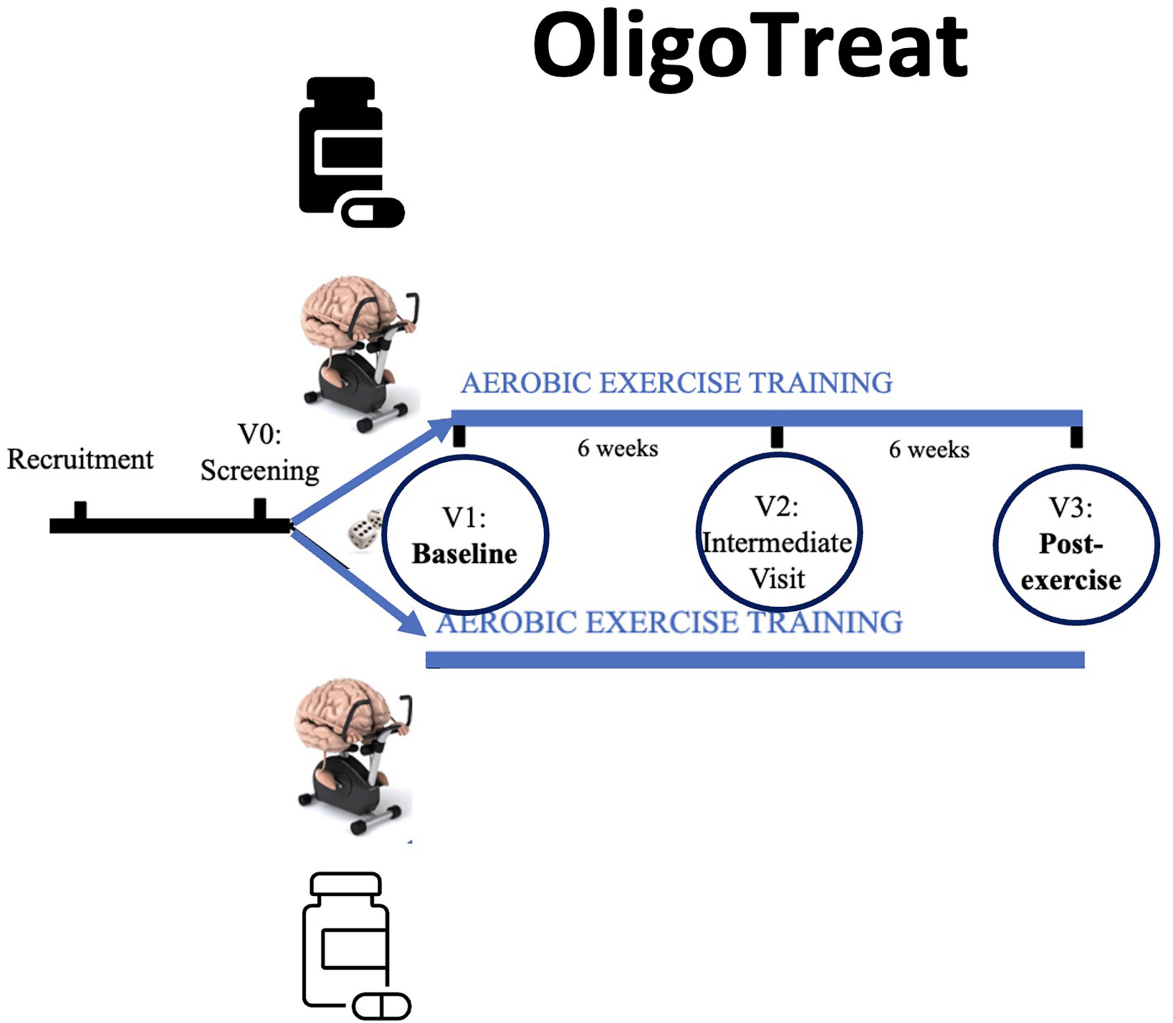
Study timeline of OT.

For further details on the assessments used, please refer to Supplementary material, particularly Supplementary Table 2. The self-constructed questionnaires are additionally provided in the Supplementary materials. The study timelines of BT and OT are shown in Figures 1, 2.

Training adherence was recorded at each session, capturing the date, duration of training (in minutes), and, if applicable, the reason for absence. Reasons for missed exercise sessions were documented based on participants’ reports. If participants did not attend to a scheduled session, they were contacted by telephone and asked about their reasons for absence. Vacation periods were reported in advance and were prospectively recorded in the study documentation. To distinguish between participant-related absences and absences due to external constraints, reasons for absence were classified into motivation-related and non-motivation-related categories using predefined categories. Non-motivation-related absences comprised unavoidable external reasons that prevented participation (e.g., illness, vacation, or other external obligations). In contrast, motivation-related absences referred to missed sessions not attributable to external constraints, where participation was considered possible. To enhance reliability, absence classification was independently performed by both trainers involved in the intervention, with discrepancies resolved through discussions and consensus. For the calculation of adherence, sessions that did not take place as scheduled and absences due to non-motivation-related reasons were excluded from the total number of possible sessions. Adherence was calculated for all participants who entered the intervention phase as the percentage of attended sessions relative to the remaining number of possible sessions. Participants contributed adherence data until withdrawal or until intervention completion.

### 2.4 Statistical analysis

Statistical analyses were performed using RStudio (Version 2026.01.0+392). The characteristics of the study population, including age, sex, years of education, disorder duration and body mass index (BMI), were analyzed using descriptive statistics and are presented in detail in the Results section.

Ten separate multiple linear regression models were estimated in this study. Model 1–6 examined predictors of training adherence in confirmatory analyses, whereas models 7–9 examined additional predictors of training adherence in exploratory analyses. Model 10 examined personality traits as predictors of PWC as a secondary exploratory outcome.

(1) Intrinsic motivational factors

(2) Personality traits,

(3) Fitness,

(4) Previous exercise experience and emotional support,

(5) Symptom severity (PANSS subscales),

(6) Symptom severity (PANSS total score),

(7) Functioning in daily life,

(8) Affective Forecasting,

(9) Satisfaction with exercise training,

(10) Personality traits as predictors of PWC.

Model 2 examined all five personality traits as predictors of training adherence, although hypotheses focused on extraversion, agreeableness and conscientiousness. This approach allowed for a comprehensive assessment.

Data from the two randomized controlled trials (BT and OT) were pooled for the analyses to investigate the overall associations across a harmonized study framework. Both trials applied comparable study designs and intervention structures. To assess the robustness of the findings with respect to the potential between-study heterogeneity, sensitivity analyses were performed by including the study origin (BT vs. OT) as a covariate in the regression models. Models containing variables assessed exclusively within the BT cohort were excluded. These analyses examined whether the observed effects were influenced by differences between the two parent trials. Other potential sources of heterogeneity (intervention arm, medication condition, and training type) were not included as covariates due to limited subgroup sizes, which precluded stable estimation of additional parameters.

An additional sensitivity analysis examined the robustness of the findings with respect to the operationalization of adherence. For these analyses, adherence was recalculated by including all absences in the denominator except pre-planned vacation periods, given their predictable and structured determined nature. Sensitivity analyses were considered exploratory and were not included in the multiple-comparison correction framework. Additional information on the sensitivity analyses is provided in the Supplementary material.

Results were considered statistically significant at a *p* < 0.05. To correct for multiple testing, the Benjamini–Hochberg method was applied to the *p*-values of the overall regression models predicting training adherence (Models 1–9), including both specifications of symptom severity. False discovery rate correction was performed at the model level, rather than at the level of individual predictor coefficients. The exploratory regression model predicting PWC (Model 10) was not included in this correction, as it represented a separate secondary outcome with a distinct analytical focus from the primary adherence analyses. Missing data were handled using listwise deletion, which was applied to the regression analyses.

## 3 Results

### 3.1 Demographic characteristics

A total of 100 patients with schizophrenia spectrum disorder (according to ICD10 (Bundesinstitut für Arzneimittel und Medizinprodukte (BfArM), 2023) and DSM V (Regier et al., 2013) were recruited at study centers of the LMU Hospital in Munich. Out of the 100 participants, 24 did not meet inclusion criteria at screening (16 BT, 8 OT), 12 participants were dropped out (9 BT, 3 OT). The participants’ characteristics are shown in Table 1.

**TABLE 1:** Participant characteristics.

Characteristics	*N*	Mean (SD)
Full sample	100 (71 BT, 29 OT)	
After exclusion of screening failures	76 (55 BT, 21 OT)	
Age (years)	76	37.90 (11.20)
Male	40 (52.60%)	
Female	36 (47.40%)	
Years of education	76	16.90 (3.77)
Disorder duration (months)	76	126.00 (114.00)
BMI	76	28.80 (5.10)

*N* denotes the number of observations; Mean and SD refer to mean values and standard deviations, respectively. BMI, body mass index; BT, BrainTrain; OT, OligoTreat; SD, standard deviation.

### 3.2 Assessments and outcomes

**FIGURE 3:**
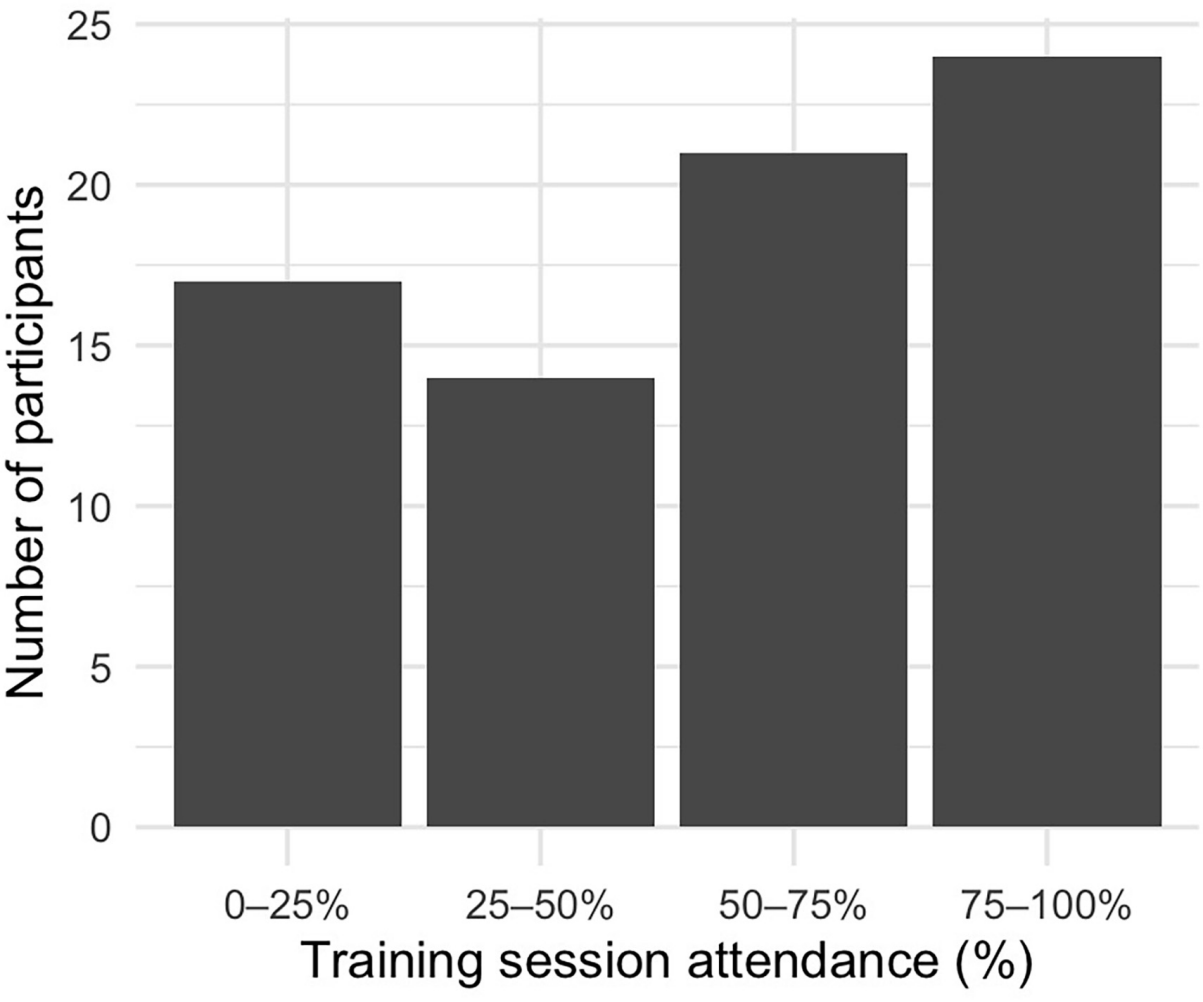
Distribution of training session attendance among participants (*N* = 76).

The primary variable of interest was training adherence, with participants completing an average of 56.3 % of offered training sessions, including a dropout rate of 15.8 %. Figure 3 illustrates the distribution of training sessions among the participants.

The secondary outcome variable of interest was fitness level, operationalized as PWC, with a mean value of 1.15 W/kg. Table 2 presents the descriptive statistics (means and standard deviations) for all study variables. The effective sample size varied across analyses (range: *N* = 41–76), due to missing data on specific variables. Table 3 reports the sample sizes and model-level regression results for each analysis. Full regression coefficients for all predictors are provided in Supplementary Table 3, and sensitivity analyses are reported in the Supplementary Tables 4, 5.

**TABLE 2:** Descriptive statistics.

Variable	*N*	Mean	SD
Training adherence	76	56.26 %	30.65
Motivation			
IE-4	64	3.55	0.91
SSA	64	4.26	0.90
EMI-2	64	3.23	0.78
Personality traits			
Extraversion	65	2.85	0.84
Agreeableness	65	3.54	0.81
Conscientiousness	65	3.35	0.73
Neuroticism	65	2.99	0.84
Openness	65	3.55	1.04
Fitness			
PWC	76	1.15	0.51
Previous exercise experience and emotional support			
Sports club during childhood	52	0.58	0.50
Sports club during youth	53	0.45	0.50
Emotional support	50	1.80	1.47
Symptom severity			
PANSS positive	76	10.83	3.44
PANSS negative	76	13.61	4.90
PANSS general	76	24.95	5.22
PANSS total	76	49.38	10.17
Functioning in daily life			
GAF	75	57.64	10.98
Affective Forecasting			
Quality of life	52	3.44	1.16
Physical health	48	3.40	1.16
Functioning	52	3.33	1.18
Cognition	52	3.25	1.19
Clinical symptoms	52	3.27	1.17
Brain function	48	3.31	1.26
Satisfaction with exercise training	41	49.17	4.47

EMI-2 composite score was based on selected subscales: challenge, competition, enjoyment, nimbleness, positive health, revitalization, strength and endurance, stress management. IE-4 was based on internal control beliefs. *N* denotes the number of available observations for each variable. Sample sizes vary across measures due to missing data. Mean and SD refer to mean values and standard deviations, respectively. EMI-2, Exercise Motivation Inventory; GAF, Global Assessment of Functioning; IE-4, Internal-External Control Beliefs Scale -4; PANSS, positive and negative syndrome scale; PWC, physical working capacity; SSA, self-efficacy toward physical exercise.

**TABLE 3:** Regression analyses.

Model	*N*	*F*	R^2^	Adjusted R^2^	*P*	Adjusted *p*
Multiple linear regression models predicting training adherence						
(1) Internal motivation	62	(3, 58) = 0.17	0.009	−0.043	0.919	0.919
(2) Personality traits	65	(5, 59) = 0.95	0.074	−0.004	0.458	0.895
(3) Fitness	76	(1, 74) = 7.2	0.089	0.076	0.009	0.081
(4) Previous exercise experience and emotional support	49	(3, 45) = 0.25	0.016	−0.049	0.860	0.919
(5) Symptom severity (PANSS subscales)	76	(3, 72) = 0.45	0.018	−0.022	0.717	0.919
(6) Symptom severity (PANSS total score)	76	(1, 74) = 0.47	0.006	−0.007	0.497	0.895
(7) Functioning in daily life	75	(1, 73) = 0.10	0.001	−0.012	0.751	0.919
(8) Affective Forecasting	48	(6, 41) = 2.33	0.254	0.145	0.050	0.225
(9) Satisfaction with exercise training	41	(1, 39) = 0.52	0.013	−0.012	0.477	0.895
Multiple linear regression model predicting PWC						
(10) Personality traits as predictors of PWC	65	(5, 59) = 0.38	0.032	−0.051	0.858	

Benjamini-Hochberg correction was applied to Models 1–9; Model 10 was excluded as a separate outcome. *N* denotes the effective sample size included in each regression model. Due to missing data on specific variables, sample sizes varied across analyses. *F* denotes the overall model test statistic. R^2^ and adjusted R*^2^* represent the coefficient of determination and adjusted coefficient of determination, respectively. Uncorrected *p values (p)* and Benjamini-Hochberg adjusted *p*-values (adjusted p) are reported.

None of the regression models remained statistically significant after correction for multiple comparison (all adjusted *p* > 0.05), including models for intrinsic motivation, personality traits, previous exercise experience and emotional support, symptom severity using the PANSS subscales, symptom severity using PANSS total score, functioning in daily life, and satisfaction with exercise training. The exploratory model combining personality traits and fitness was also not significant.

Two models were significant before correction for multiple testing: Model 3 (the fitness model), *F* (1, 74) = 7.2, *R^2^* = 0.089, *adjusted R^2^* = 0.076, *p* = 0.009, and Model 8 (the affective forecasting model), *F* (6, 41) = 2.33, *R^2^* = 0.254, *adjusted R^2^* = 0.145, *p* = 0.050.

Neither remained significant after Benjamini-Hochberg correction: Model 3 (the fitness model) *adjusted p* = 0.081, and Model 8 (the affective forecasting model) *adjusted p* = 0.225.

## 4 Discussion

The present study aimed to gain a deeper understanding of psychological, clinical and behavioral factors related to training adherence in individuals with schizophrenia during a structured physical exercise intervention. Specifically, we examined whether intrinsic motivation, selected personality traits, fitness levels, previous exercise experience, symptom severity, effectiveness expectations and satisfaction with the intervention were associated with adherence. In addition, we explored whether personality-related variables were associated with physical fitness. Contrary to the hypothesized associations, the analyses did not reveal robust associations between the investigated variables and the outcomes.

No significant association between intrinsic motivational factors and exercise adherence was observed in the present study. While previous literature (Vancampfort et al., 2013; Costa et al., 2018) has reported associations between intrinsic motivational factors and exercise behavior, the present findings did not support this pattern. A large cross-sectional online survey using the EMI and SSA questionnaires indicated that intrinsic motivation and exercise-related self-efficacy expectations were key correlates of higher physical activity in individuals with mental illness (Koepl et al., 2025), but the results did not divide into diagnostic subgroups making conclusions for people with schizophrenia difficult. Differences in measurement approaches may partly explain these discrepancies. Whereas previous research has largely relied on self-reported questionnaires, which may be subject to reporting bias and overestimation of activity levels, the present study used objectively recorded training attendance as the primary outcome measure.

Contrary to expectations, no significant associations were observed between the Big Five personality traits (extraversion, conscientiousness, agreeableness, openness, and neuroticism) and exercise adherence. In particular, the personality traits extraversion and conscientiousness have been identified with exercise behavior in prior research (Rhodes and Smith, 2006; Müller, 2023; Caille et al., 2024). Evidence regarding agreeableness has been less consistent (Caille et al., 2024), although a recent large cross-sectional study demonstrated an association between agreeableness and regular engagement in physical activity (Wambsganz et al., 2024). Personality traits may not be robust predictors of objectively measured exercise adherence in individuals with schizophrenia, possibly reflecting differences in outcome measurement (objective vs. self-report). Moreover, exercise-related behavior in schizophrenia may be driven more strongly by clinical factors such as symptom severity, medication effects, and reduced activity levels. This may attenuate or override potential associations with stable personality traits.

Personality traits were evaluated as potential predictors of fitness. Although recent evidence in healthy individuals has suggested that extraversion and conscientiousness are associated with cardiorespiratory fitness and peak power output (Ronca et al., 2025), no significant relationships were observed between personality traits and fitness in the present sample. Individuals with schizophrenia exhibit lower levels of extraversion, conscientiousness, and agreeableness (Ohi et al., 2016). These discrepancies could reflect differences in the underlying mechanisms linking personality and physical fitness across populations and may not be applicable to individuals with schizophrenia.

Considering that individuals with schizophrenia often present with reduced cardiorespiratory fitness (Vancampfort et al., 2015), physical fitness was examined as a potential predictor of exercise adherence. No significant association was observed between fitness and exercise adherence. The present findings are consistent with Schwaiger et al. (2024), who also reported no significant relationship between baseline physical fitness and exercise adherence over the course of the study. This suggests that physical fitness is unlikely to be a contributing determinant of sustained engagement in structured exercise interventions in this population. Instead, adherence may be more strongly influenced by contextual factors that are independent of physical capacity. Additionally, although most participants remained enrolled throughout the intervention period, attendance was substantially lower than the intended training frequency. The World Health Organization (WHO) recommends at least 150 min of moderate-intensity physical activity per week (Bull et al., 2020). Individuals with severe mental disorders are less likely to meet these recommendations than healthy controls (Vancampfort et al., 2017). Since greater exercise exposure and adherence to prescribed training volumes may contribute to more pronounced intervention benefits (Firth et al., 2015; Yang et al., 2024), understanding factors influencing exercise adherence in this population remains important.

The present study did not find evidence that previous exercise experience or emotional support are associated with exercise adherence among individuals with schizophrenia. This may indicate that early-life exercise experience and perceived social support are not major determinants of current exercise behavior in this clinical population. A recent study suggested that adolescents engaging in physical activity are more likely to maintain an active lifestyle into emerging adulthood (Bélanger et al., 2025). The applicability of these findings to clinical samples remains unclear. Early-life influences may be less strongly linked to current exercise behavior in schizophrenia, where adherence is more immediately shaped by present-day factors such as treatment structure, symptom- related impairments, and reduced functional capacity. This may override distal developmental effects.

The present findings do not support a relationship between symptom severity and exercise adherence. In contrast to previous investigations, neither the baseline PANSS subscale scores nor the baseline PANSS total score were significantly associated with training participation (see Supplementary Table 3 for more details). This contrasts with earlier findings indicating that higher levels of negative symptoms are associated with reduced physical activity in individuals with schizophrenia (Vancampfort et al., 2012). This may be explained by generally low levels of negative symptoms in the present sample. Eligibility criteria restricted the sample to individuals with PANSS total scores below 75, resulting in a relatively homogeneous range of symptom severity.

High levels of daily functioning in combination with low symptom severity have previously been suggested to be associated with greater adherence to exercise programs (Schwaiger et al., 2024). In contrast, the present exploratory analysis did not identify a significant association between daily functioning and training participation. The mean GAF score in the present sample was 57, indicating moderate impairment in overall functioning (Endicott et al., 1976). This suggests a restricted range of functioning within the sample, as both functional level and symptom severity were relatively low in this study. Such reduced variability may have limited the statistical power to detect associations with exercise adherence.

Additionally, affective forecasting was examined as an exploratory predictor of exercise adherence. No significant associations between affective forecasting variables and exercise adherence were observed after correction for multiple testing, providing no evidence for an association in the present sample.

Satisfaction with the exercise intervention was additionally examined as an exploratory indicator of participants’ subjective emotional experience during the intervention. Previous findings have linked patient dissatisfaction to higher dropout rates (Schoemaker et al., 2019), whereas no significant association between satisfaction and training adherence was observed in the present study. Limited variability in satisfaction ratings may have reduced statistical power to detect associations with training adherence.

Several strengths of the present study should be highlighted. First, the study was initially based on a comparatively large sample for exercise research in individuals with schizophrenia spectrum disorders, enabling the examination of a broad range of psychological, behavioral, and clinical variables as potential predictors of exercise adherence. Adherence was assessed using objectively recorded training attendance rather than self-reported physical activity measures, thereby reducing the risk of reporting bias. This combination allowed the examination of multiple potential determinants of exercise adherence.

Moreover, the study addresses an understudied intervention approach, targeting an important research gap and offering potential clinical relevance for future exercise-based interventions. Since the study was conducted and reported in a transparent and systematic manner, replication is facilitated and the credibility of the findings is strengthened.

The intervention was delivered in a small-group setting, under the supervision of trained exercise professionals, ensuring close monitoring and providing individualized support. These intervention characteristics have been highlighted as potentially important moderators of the effectiveness of lifestyle interventions in individuals with severe mental illness (Maurus et al., 2024). Consequently, they may have enhanced participant engagement and supported high-quality intervention delivery.

Several limitations should be considered when interpreting these findings. The present study may have been limited in its ability to detect small-to-moderate associations due to variability in effective sample sizes across models (*N* = 41–76). While most models included an acceptable number of observations relative to the number of predictors, several analyses were affected by missing data, resulting in reduced statistical power. Accordingly, the analyses were likely primarily powered to detect moderate to large effects, whereas smaller effects may have been insufficiently detected. A further limitation concerns the overall variability of the study sample. The participants showed relatively homogeneous characteristics across several measures, which was partly reflected in the present dataset. This pattern was influenced to some extent by the applied inclusion criteria, which restricted participation to individuals with PANSS total scores below 75. As a result, individuals with more severe symptom profiles, including pronounced negative symptoms, were not represented, which may have reduced variability in key clinical characteristics and limited sensitivity to detect meaningful associations.

Within the clinical domain, general measures of symptom severity and functioning (i.e., PANSS and GAF) were included in the analyses. More specific clinical and treatment-related variables were not included as covariates in the present analyses. This particularly concerns variables such as antipsychotic medication type and dose, extrapyramidal symptoms, sedation, depressive symptom severity as a distinct construct, and cognitive impairment. Accordingly, more specific potentially relevant confounders were not accounted for. Contextual and environmental factors were not systematically included in the analytic models. These include socioeconomic barriers, transportation distance, employment status, social support during the intervention, as well as competing demands such as work commitments, family responsibilities, and engagement in other leisure-time physical activities. Although reasons for missed sessions were documented and categorized during the intervention, these contextual factors were not incorporated as predictors of adherence in the statistical analyses. More dynamic aspects such as day-to-day fluctuations in well-being and perceived capacity to engage in structured activity were also not captured. Taken together, this suggests that important clinical and contextual determinants of exercise adherence my not have been fully represented in the present analytical framework.

Moreover, several self-constructed questionnaires were used to assess previous experience and emotional support, affective forecasting and satisfaction with the intervention. While these instruments were developed to capture study-specific constructs not covered by standardized measures, they have not been formally validated in independent psychometric studies. Consequently, information on reliability and construct validity is limited, introducing measurement uncertainty.

A proportion of participants may not have engaged in sports prior to the study, suggesting that the initial training frequency may have been relatively demanding for individuals with little or no prior structured exercise experience. Furthermore, self-initiated physical activity in everyday life is not directly equivalent to attendance in a structured, time-constrained exercise intervention. This may have limited comparability with previous studies focusing on habitual physical activity.

Future research should explore additional strategies to improve adherence to exercise interventions in individuals with schizophrenia. The present study did not identify any statistically significant association between individual variables and exercise adherence. Further investigations should aim to replicate these findings in larger samples to further examine whether these null associations are robust and generalizable. In addition, a substantial proportion of participants in the present study did not comply with the training protocol, highlighting the need to better understand how sustained engagement in structured exercise interventions can be supported. One potential approach may be the implementation of more flexible scheduling strategies, such as adjustable training times, that allow participants to reschedule sessions or include fixed catch-up sessions (e.g., weekend sessions). This might help to reduce barriers to regular participation. Logistical support including practical assistance such as on-site childcare, app-reminders and equipment provisions (e.g., shoes), could support sustained engagement in physical activity. Moreover, extending the study duration may allow participants to gradually adapt to the recommended training frequency and thereby facilitate adherence to more frequent weekly sessions. It may also be of interest to examine whether group-based and individual training formats influence motivation, comfort and sustained engagement differently. Taken together, the present findings suggest that no single individual characteristic emerged as a robust predictor of adherence within this study. Instead, more flexible and individualized approaches may be needed to support regular physical activity among individuals with schizophrenia.

In conclusion, the present study did not identify significant predictors of exercise adherence among individuals with schizophrenia. Although some effects were in the expected directions, none remained significant after correction for multiple testing. These findings suggest that exercise adherence in this population may be less determined by stable individual characteristics and more influenced by modifiable structural and contextual factors. Consequently, intervention design may need to place greater emphasis on flexible and supportive implementation strategies to improve adherence in this population.

## The BrainTrain Working Group

Amann M., Bauerreiß J., Diegelmann J., Glad J., Jannan J., John M., Kara E. B., Koc Ö., Lada K., Moussiopoulou J., Sagstetter L., Sautner J., Segerer J., Theis N., Wartensleben von J., Weibel A., Widmann L., Yakimov V., Yildrim D., Zuliani M.

## Data Availability

The data analyzed in this study is subject to the following licenses/restrictions: Due to privacy and ethical restrictions, the underlying raw data are not publicly available. Requests to access these datasets should be directed to Susanne.kuprat@med.uni-muenchen.de.
