# Supplementary material for: Individual factors associated with physical activity adherence in schizophrenia: an exploratory secondary analysis of two randomized controlled trials
Source: Front Psychol. 2026 Jul 28;17:1878217. doi: 10.3389/fpsyg.2026.1878217 (PMC13457105; doi:10.3389/fpsyg.2026.1878217)
Supplement: Supplementary file 1 [file Data_Sheet_1.pdf]

Table S1. Inclusion and exclusion criteria of BrainTrain and OligoTreat

**BrainTrain**		
	**Inclusion**	**Exclusion**
**Participants**	Patients diagnosed with schizophrenia, both inpatients and outpatients, as per ICD-10 and DSM-5 criteria, confirmed through the Mini-International Neuropsychiatric Interview (M. I. N. I. Version 6.0.0);<br><br>Written informed consent for voluntary participation in the study.	Acute suicidal behavior or risk to others;<br>Significant somatic or neurological comorbidities;<br>Other major Axis I psychiatric disorders;<br>Other pertinent neurological or related disorders, or lack of reliability and sanity (as assessed by an independent psychiatrist).
**Age**	18 to 65 years	
**Gender**	Female, male	
**PANSS**	PANSS total at baseline ≤ 75	
**Medication and drugs**	A stable psychopharmacological regimen for a minimum of two weeks;<br>All forms of antipsychotic substances, including depot medications;<br>Adjunctive antipsychotics for agitation or sleep disturbances, provided the total daily dosage did not exceed 150 mg in chlorpromazine equivalents.	Positive urine drug screen for illicit substances or cannabis (excluding benzodiazepines)
**Women of childbearing age**	Proper contraception	Pregnancy;<br>Lactation.
**Language**	German language skills	Incapacity to provide informed consent or inadequate proficiency in the German language.

**OligoTreat**		
	**Inclusion**	**Exclusion**
**Participants**	The diagnosis of schizophrenia or schizophrenia spectrum disorder based on the DSM-V criteria through the M. I. N. I. interview;<br><br>Written informed consent.	Clinically significant central nervous system (CNS) disorders (such as epilepsy or a history of seizures);<br>The presence of unstable somatic conditions, such as porphyria, narrow-angle glaucoma, stenosing peptic ulcer, pyloroduodenal obstruction, or prostatic hypertrophy with urinary retention and bladder neck obstruction;<br>Hereditary galactose intolerance, lapp lactose deficiency, or glucose-galactose malabsorption;<br>Clinically relevant hepatic or renal impairment, cardiac comorbidities, current hypokalemia, and clinically relevant hyponatremia at screening;<br>Known intolerance, allergy, or contraindications to any of the study drugs, their excipients, or agents with similar chemical properties, such as other arylalkylamine antihistamines;<br>Enrollment in another clinical trial involving an investigational medicinal product or participation within the last 30 days or 7 half-lives of a previously used investigational medicinal product;<br><br>For optional MRI assessment: any potential contraindications to MRI.
**Age**	18 to 65 years	
**Gender**	Female, male	
**PANSS**	PANSS score </= 75 at VO	
**Medication and drugs**	A stable dose of antipsychotic treatment for a minimum of one week prior to inclusion;<br>Stability of any CNS-active treatment substances and their dosages for at least one week prior to inclusion;	Coercive treatment at the time of study inclusion;<br>Concomitant use of any other potential remyelinating therapy;<br>Current treatment with agents with strong anticholinergic properties (such as MAO inhibitors, opioid antagonists, or clozapine), with macrolide antibiotics, (such as erythromycin or clarithromycin, or azole-type antimycotics).

**Women of childbearing age**	A negative serum beta-hCG pregnancy test (as part of the screening process), including a negative test within seven days prior to randomization;<br>Incapable of childbearing or using medically approved method of contraception for the entire duration of the study.	Pregnancy;<br>Breast-feeding.
**Language**	German language skills	Incapacity to provide informed consent or inadequate proficiency in the German language.

Note. Abbreviations: CNS, Central Nervous System; DSM-5, Diagnostic and Statistical Manual of Mental Disorders (5th ed.); hCG, human chorionic gonadotropin; ICD-10, International Classification of Diseases (10th rev.); M. I. N. I., Mini-International Neuropsychiatric Interview; PANSS, Positive and Negative Syndrome Scale; V0, baseline assessment.

Table S2. Details and timetable of the used questionnaires

	Abbreviations	Details	Time	Note
**Motivation**				
Internal-External control beliefs	IE-4	The IE-4 scale measures internal and external locus of control.	V0	
Exercise Motivation Inventory	EMI-2	The EMI-2 categorizes exercise motives into intrinsic domains (challenge, competition, enjoyment, nimbleness, positive health, revitalization, strength and endurance, stress management) and extrinsic domains (affiliation, appearance, ill-health avoidance, social pressure, social recognition, weight management).	V0	
Self-efficacy towards physical exercise	SSA	The SSA scale measures self-efficacy for exercising under challenging conditions.	V0	
**Personality traits**				
Big five inventory	BFI-10	The BFI-10 assesses the five personality traits: openness, conscientiousness, extraversion, agreeableness, and neuroticism	V0	
**Fitness**				
Physical Working Capacity 130	PWC 130	The workload corresponding to a heart rate of 130 beats per minute.	V0	
**Previous experiences with exercise**		Study-specific anamnesis questionnaire assessing early-life physical activity, sports participation, and family support.	V0	BrainTrain

**Clinical symptoms**				
Positive and Negative Syndrome Scale	PANSS	Positive, negative, general and total symptom scores in schizophrenia.	V1	
**Functioning in daily life**				
Global Assessment of Functioning	GAF	A measure of overall psychological, social, and occupational functioning.	V1	
**Effectiveness**				
Affective forecasting		Study-specific individual assessment of sports intervention effectiveness compared to others; Including: clinical symptoms, cognitive performance, general level of functioning, quality of life, physical health, and brain structure and function.	V1	BrainTrain
**Satisfaction**				
Satisfaction with exercise training		A study-specific measure;<br>Participants' subjective experiences and satisfaction with different aspects of the exercise intervention.	V2	BrainTrain

Note. Measures marked with BrainTrain were not available in the OligoTreat (OT) cohort. Abbreviations: BFI-10, Big Five Inventory; EMI-2, Exercise Motivation Inventory-2; GAF, Global Assessment of Functioning; IE-4, Internal-External Control Beliefs Scale; PANSS, Positive and Negative Syndrome Scale; PWC130, Physical Working Capacity at 130 beats/ minute; SSA, Self-efficacy towards physical exercise; V0, baseline assessment; V1, assessment day 0–7; V2, assessment day 30–37.

Additional information concerning the assessments is provided.

IE-4 scale

The IE-4 scale offers a concise assessment of internal and external locus of control through four items and employs a five-point Likert response format. Grounded in Rotter's social learning theory, the scale was assessed using two extensive German samples. Verified by confirmatory factor analysis, the scale reveals a stable two-factor structure. The observed results were largely consistent with the locus of control theory. The reliability is adequate for group-level analyses, exhibiting moderate internal consistency (McDonalds's ω = .53-.71) and retest stability (*rtt* = .56-.64) (Kovaleva et al., 2012).

EMI-2

The Exercise Motivations Inventory (EMI), developed by Markland and Hardy (1993), and based on Self-Determination Theory, aims to evaluate exercise motives that influence sustained physical activity. Initially, the inventory was formulated based on open-ended responses and a comprehensive literature review, resulting in 71 items. Redefining these items resulted in 44, distributed across 12 factors, through factor analysis. In 1997, Markland and Ingledew revised the inventory, producing the EMI-2, which comprises 51 items and 14 subscales. Applicability was extended to both, exercisers and non-exercisers. The scale exhibits strong internal consistency, with Cronbach's alpha values ranging from .69 to .95. It demonstrates satisfactory test–retest reliability, with coefficients approximately between r = .70 and r = .90 across all subscales. A German adaption was developed for research involving adolescents.

SSA:

Fuchs and Schwarzer (1994) introduced a 12-item scale, designed to assess self-efficacy for exercising under challenging conditions. This scale, known as the SSA scale, was tested in a large population sample. It demonstrates a high level of internal consistency ($\alpha \approx .89$). Construct validity is supported by its associations with general self-efficacy, exercise intentions, and its negative correlations with psychological distress. Longitudinal studies have affirmed the scale's predictive validity, demonstrating its suitability for predicting future physical activity behavior.

BFI-10

The BFI-10 evaluates the five personality traits of extraversion, conscientiousness, agreeableness, openness, and neuroticism. Each dimension is assessed using a five-point rating scale with two items per trait (one positively keyed and one negatively keyed). Test-retest reliability is considered acceptable for group comparison (rtt =.49 to .84). Factor analyses on heterogeneous samples confirmed the expected five-factor structure. Theoretically consistent findings, including correlations with the NEO Personality Inventory, support the construct validity. This indicates convergent measurement of the Big Five traits. Associations with external criteria (e.g., health and social behavior indicators) follow the expected patterns, confirming that the personality constructs are adequately captured (Rammstedt et al., 2013).

PANSS

The Positive and Negative Syndrome Scale is internationally recognized as the gold standard for evaluating schizophrenia symptoms. Interrater reliability is supported by an intraclass correlation coefficient (*ICC)* of .80. Internal consistency coefficients for the positive,

negative, and general psychopathology subscales are acceptable, with coefficients ranging from $\alpha \approx .73$ to .83. Significant correlations with established clinical measurements support criterion validity. Construct validity was evidenced by the inverse relationship between the positive and negative symptom scales (Kay et al., 1987).

GAF

The Global Assessment of Functioning scale is used to assess the overall psychological, occupational and social functioning of patients with psychiatric disorders in their daily life. The scale spans from 0 to 100, structured in ten-point intervals. Reliability coefficients range from $r = .56$ to $r = .76$, contingent on model specification and the inclusion of rater effects. Interrater variability is observed, with relative bias estimates of up to approximately 18-23 points. Criterion validity is demonstrated through significant correlations between GAF scores and the patients' required level of support, as well as their adjustment to medication (Jones et al., 1995).

Affective Forecasting

Wilson and Gilbert (2003) described affective forecasting as the process of predicting one's emotional responses to future situations. Individuals often overestimate the emotional impact of future events. This phenomenon is known as impact bias, resulting from a failure to account for psychological adaptation. Participants' expectations regarding the potential benefits of their assigned exercise intervention were evaluated using a study-specific Affective Forecasting Questionnaire (developed for the BT trial). They were asked to compare their assigned intervention with the alternative exercise program. Items addressed anticipated effects on future clinical symptoms, overall daily functioning, quality of life, physical health,

and changes in brain structures and function. For instance, participants rated the item: *”Compared with the other exercise intervention, how much do you expect to benefit from your exercise program with regard to your current clinical symptoms?”* Responses were recorded on a six-point Likert-type scale ranging from *“I will not benefit from either exercise program”* to *“I will benefit substantially more from my assigned exercise program.”* The instrument was not based on a standardized or psychometrically validated scale.

Satisfaction with exercise training

Satisfaction with exercise training is defined as the degree of gratification an individual experiences when engaging in exercise (Kim et al. 2024). In the present study, this construct was evaluated using Items 1-12 from a study-specific, self-constructed questionnaire (developed for the BT trial), which was not based on a standardized or psychometric validated scale. The questionnaire captured the participants’ subjective experiences and satisfaction with various aspects of the exercise intervention. Example items included: *“The training was easy to integrate into my daily routine”* and *“I felt well supported during the training sessions.”* Responses were recorded on a five-point Likert scale ranging from *“not at all”* to *“very much”.*

Previous exercise experience and emotional support

Previous exercise experience and emotional support was assessed using a qualitative, study-specific questionnaire (developed for the BT trial). This instrument gathers retrospective data on early life physical activity and participation in organized sports as well as perceived emotional support for sport participation within the family context. Example items included: *“Were you a member of a sports club during childhood (before the age of 12)?”,* and *“Did*

your parents or family provide emotional support for your sporting activities during childhood or adolescence, for example through encouragement?". The items were either coded as dichotomous variables (0= no, 1= yes) or rated on a five-point Likert scale ranging from "not at all" to "very much". The questionnaire was not based on a standardized or psychometrically validated scale.

Affective Forecasting

Einschätzung der Wirksamkeit der Sportprogramme

Mit den folgenden Fragen interessieren wir uns für Ihre Einschätzung über die Wirksamkeit der beiden Sportinterventionen, die im Rahmen der BrainTrain-Studie durchgeführt werden. Wie Sie wissen absolviert eine Gruppe ein aerobes Ausdauersportprogramm auf einem Fahrradergometer, während die andere Gruppe ein Beweglichkeits-, Gleichgewichts- und Krafttraining durchläuft. Sie wurden nach dem Zufallsprinzip einer dieser beiden Gruppen zugeteilt. Nun möchten wir wissen, wie Sie die **Wirksamkeit Ihrer Sportintervention im Vergleich zur anderen** einschätzen. Es geht dabei **nicht** darum, die allgemeine Wirksamkeit beider Interventionen zu beurteilen, sondern lediglich **um Ihre Einschätzung**, welche der beiden Interventionen **Ihnen ganz persönlich** besser helfen würde. Beispielsweise könnten Sie dem aeroben Ausdauersportprogramm zugeteilt worden sein, obwohl Sie eigentlich lieber das Beweglichkeits-, Gleichgewichts- und Krafttraining gemacht hätten, da diese Art von Sport Ihnen in der Vergangenheit für Ihr Wohlbefinden besser geholfen hat. Natürlich könnte es auch anders herum sein. Genau das möchten wir mit den folgenden Fragen herausfinden. Es gibt dabei keine richtigen oder falschen Antworten. Es geht lediglich um **Ihre persönliche Einschätzung**.

1) Wie stark werden Sie von Ihrem Sportprogramm im Vergleich zur anderen Sportintervention bezüglich Ihrer aktuellen **klinischen Symptome** profitieren?

○	○	○	○	○
Ich werde deutlich weniger profitieren	Ich werde etwas weniger profitieren	Ich werde genauso profitieren	Ich werde etwas mehr profitieren	Ich werde deutlich mehr profitieren

○ Meine klinischen Symptome werden durch keines der beiden Sportprogramme besser werden

2) Wie stark werden Sie von Ihrem Sportprogramm im Vergleich zur anderen Sportintervention bezüglich Ihrer **kognitiven Leistungsfähigkeit** profitieren?

○ Ich werde deutlich weniger profitieren
○ Ich werde etwas weniger profitieren
○ Ich werde genauso profitieren
○ Ich werde etwas mehr profitieren
○ Ich werde deutlich mehr profitieren

○ Meine kognitive Leistungsfähigkeit wird durch keines der beiden Sportprogramme besser werden

3) Wie stark werden Sie von Ihrem Sportprogramm im Vergleich zur anderen Sportintervention bezüglich Ihrem **allgemeinen Funktionsniveau** profitieren?

○ Ich werde deutlich weniger profitieren
○ Ich werde etwas weniger profitieren
○ Ich werde genauso profitieren
○ Ich werde etwas mehr profitieren
○ Ich werde deutlich mehr profitieren

○ Meine allgemeines Funktionsniveau wird durch keines der beiden Sportprogramme besser werden

4) Wie stark werden Sie von Ihrem Sportprogramm im Vergleich zur anderen Sportintervention bezüglich Ihrer **Lebensqualität** profitieren?

○ Ich werde deutlich weniger profitieren
○ Ich werde etwas weniger profitieren
○ Ich werde genauso profitieren
○ Ich werde etwas mehr profitieren
○ Ich werde deutlich mehr profitieren

○ Meine Lebensqualität wird durch keines der beiden Sportprogramme besser werden

5) Wie stark werden Sie von Ihrem Sportprogramm im Vergleich zur anderen Sportintervention bezüglich Ihrer **körperlichen Gesundheit** profitieren?

○ Ich werde deutlich weniger profitieren
○ Ich werde etwas weniger profitieren
○ Ich werde genauso profitieren
○ Ich werde etwas mehr profitieren
○ Ich werde deutlich mehr profitieren

○ Meine körperliche Gesundheit wird durch keines der beiden Sportprogramme besser werden

6) Wie stark werden Sie von Ihrem Sportprogramm im Vergleich zur anderen Sportintervention bezüglich Ihrer **Gehirnstrukturen und -funktionen** profitieren?

O	O	O	O	O
Ich werde deutlich weniger profitieren	Ich werde etwas weniger profitieren	Ich werde genauso profitieren	Ich werde etwas mehr profitieren	Ich werde deutlich mehr profitieren

O Meine Gehirnstrukturen und –funktionen werden sich durch keines der beiden Sportprogramme verändern

Proband: BT__________ Untersucher: _________________ Datum: _________________

Zufriedenheit mit dem Training im Rahmen der Studie

Die Räumlichkeiten für das Training sind angemessen

□ überhaupt nicht □ kaum □ neutral □ ein bisschen □ sehr

Die Geräte für das Training sind angemessen

□ überhaupt nicht □ kaum □ neutral □ ein bisschen □ sehr

Das Training war zeitlich gut in meinen Alltag zu integrieren.

□ überhaupt nicht □ kaum □ neutral □ ein bisschen □ sehr

Im Training habe ich mich gut betreut gefühlt.

□ überhaupt nicht □ kaum □ neutral □ ein bisschen □ sehr

Das Training hat mein Wissen über Sport verbessert.

□ überhaupt nicht □ kaum □ neutral □ ein bisschen □ sehr

Durch das Training fühle ich mich in meinem Körper wohler.

□ überhaupt nicht □ kaum □ neutral □ ein bisschen □ sehr

Das Training hilft mir meine Gesundheit zu verbessern.

□ überhaupt nicht □ kaum □ neutral □ ein bisschen □ sehr

Es kostet mich Überwindung, ins Training zu gehen

□ überhaupt nicht □ kaum □ neutral □ ein bisschen □ sehr

Das Training macht mir Spaß

□ überhaupt nicht □ kaum □ neutral □ ein bisschen □ sehr

Durch das Training sehe ich Fortschritte

□ überhaupt nicht □ kaum □ neutral □ ein bisschen □ sehr

Nachdem Training fühle ich mich gut

□ überhaupt nicht □ kaum □ neutral □ ein bisschen □ sehr

Das Training motiviert mich auch für mehr Bewegung im Alltag

□ überhaupt nicht □ kaum □ neutral □ ein bisschen □ sehr

Proband: BT___________ Untersucher: __________________ Datum: __________________

Die Anzahl der Trainingsteilnehmer war:

□ zu hoch □ zu gering

Die Betreuung durch das Trainingspersonal war:

□ zu hoch □ zu gering

Die Trainingsintensität war:

□ zu hoch □ zu gering

Weitere Anmerkungen zum Training:

Proband: BT__________ Untersucher: _________________ Datum: _________________

Sportanamnese:

Waren Sie als Kind (vor dem 12. Lebensjahr) Mitglied in einem Sportverein?

□ ja □ nein

Wie oft pro Woche haben Sie als Kind durchschnittlich (außerhalb des Schulsports) Sport gemacht? _______x/_Woche

Welche Note hatten Sie meist im Fach Sport im Zeugnis bis zum 12. Lebensjahr? _________

Welche Note hatten Sie meist im Fach Sport zwischen dem 12. und 18. Lebensjahr? _______

Waren Sie als Jugendliche/r (12.- 18. Lebensjahr) Mitglied in einem Sportverein?

□ ja □ nein

Wie oft pro Woche haben Sie durchschnittlich als Jugendliche/r (außerhalb des Schulsports) Sport gemacht? _______x/_Woche

Haben Sie in ihrer Kindheit/Jugend eine oder mehrere Sportarten ausgeübt, aber wieder aufgehört? Falls ja:

□ Kein Spaß

□ Kein Talent/keine Erfolgsmomente

□ Nicht mit anderen Kindern verstanden

□ Nicht mit Trainer verstanden

□ Zu anstrengend

□ Zu großer Erfolgsdruck

□ Zu zeitraubend

□ Andere Hobbies wichtiger

□ Schule/Ausbildung wichtiger

□ Finanzielle Gründe

□ Anderes

Hätten Sie gerne als Kind/Jugendlicher eine Sportart ausgeübt, hatten aber keine Gelegenheit dazu?

□ Nein

□ Ja – Kein Angebot in der Nähe

□ Ja – Finanzielle Gründe

□ Ja – keine familiäre Unterstützung

□ Ja – keine Zeit

□ Anderes

Proband: BT___________ Untersucher: __________________ Datum: __________________

Haben Ihre Familienmitglieder während Ihres Aufwachsens regelmäßig (mind. 1x pro Woche) Sport gemacht?

□ Nein

□ Ein Elternteil

□ Beide Elternteile

□ Geschwister

Haben Ihre Eltern/ Ihre Familie Ihre sportliche Aktivität als Kind/Jugendlicher **emotional** z.B. durch Ermutigung unterstützt?

□ überhaupt nicht □ kaum □ neutral □ ein bisschen □ sehr

Haben Ihre Eltern/ Ihre Familie Ihre sportliche Aktivität als Kind/Jugendlicher **durch Informationen** (z.B. über Angebote) unterstützt?

□ überhaupt nicht □ kaum □ neutral □ ein bisschen □ sehr

Haben Ihre Eltern/ Ihre Familie Ihre sportliche Aktivität als Kind/Jugendlicher **instrumentell** unterstützt (z.B. durch Fahrten zum Training oder zu Wettkämpfen)? □ überhaupt nicht □ kaum □ neutral □ ein bisschen □ sehr

Sind Sie aktives Mitglied in einem Sportverein?

□ ja □ nein

Sind Sie Mitglied in einem Fitnessstudio?

□ ja □ nein

Sind Sie Mitglied in einer freien Trainingsgruppe? (z.B. Walkinggruppe)?

□ ja □ nein

Nutzen Sie kostenlose Sportangebote (z.B. Sport im Park)?

□ ja □ nein

Ich treibe Sport meist... **Am liebsten treibe ich Sport....**

□ alleine

□ mit Familienmitgliedern

□ mit Freunden

□ in einer festen Gruppe.

□ in einer wechselnden Gruppe.

□ alleine

□ mit Familienmitgliedern

□ mit Freunden

□ in einer festen Gruppe.

□ in einer wechselnden Gruppe

Table S3. Multiple regression results for all outcome models

Multiple linear regression models predicting training adherence

Model 1: Internal motivation

$N = 62$, $F(3, 58) = 0.17$, $R^2 = .009$, *adjusted* $R^2 = -.043$, *p (model)* = .919, *adjusted p* = .919,

Predictor	*B*	*SE*	*t*	*p (predictor)*
**IE-4**	-0.84	4.61	-0.18	.856
**SSA**	-0.24	4.41	-0.06	.957
**EMI-2**	3.55	5.08	0.70	.487

Model 2: Personality traits

$N = 65$, $F(5, 59) = 0.95$, $R^2 = .074$, *adjusted* $R^2 = -.004$, *p (model)* = .458, *adjusted p* = .895

Predictor	*B*	*SE*	*t*	*p (predictor)*
**Extraversion**	-2.25	4.88	-0.46	.646
**Agreeableness**	6.44	4.81	1.34	.186
**Conscientiousness**	-4.92	5.31	-0.93	.357
**Neuroticism**	4.10	4.62	0.89	.379
**Openness**	5.95	3.87	1.54	.130

Model 3: Fitness

N = 76, *F* (1, 74) = 7.2, R^2 = .089, *adjusted* R^2 = .076, *p (model)* = .009, *adjusted p* = .081

**Predictor**	***B***	***SE***	***t***	***p (predictor)***
**PWC**	18.00	6.71	2.68	.009

Model 4: Previous exercise experience and emotional support

N = 49, *F* (3, 45) = 0.25, R^2 = .016, *adjusted* R^2 = -.049, *p (model)* = .860, *adjusted p* = .919

**Predictor**	***B***	***SE***	***t***	***p (predictor)***
**Sports club during childhood**	-1.53	10.51	-0.15	.885
**Sports club during adolescence**	9.51	11.20	0.85	.400
**Emotional support**	-1.24	3.57	-0.35	.730

Model 5: Symptom severity (PANSS subscales)

N = 76, *F* (3, 72) = 0.45, R^2 = .018, *adjusted* R^2 = -.022, *p (model)* = .717, *adjusted p* = .919

**Predictor**	***B***	***SE***	***t***	***p (predictor)***
**PANSS positive**	0.89	1.25	0.72	.477
**PANSS negative**	-0.12	0.81	-0.16	.877
**PANSS general**	-0.86	0.89	-0.97	.337

Model 6: Symptom severity (PANSS total score)

N = 76, *F* (1, 74) = 0.47, R^2 = .006, *adjusted* R^2 = -.007, *p (model)* = .497, *adjusted p* = .895

**Predictor**	***B***	***SE***	***t***	***p (predictor)***
**PANSS total score**	-0.24	0.35	-0.68	.497

Model 7: Functioning in daily life

N = 75, *F* (1, 73) = 0.10, R^2 = .001, *adjusted* R^2 = -.012, *p (model)* = .751, *adjusted p* = .919

**Predictor**	***B***	***SE***	***t***	***p (predictor)***
**GAF**	0.11	0.33	0.32	.751

Model 8: Affective Forecasting

N = 48, *F* (6, 41) = 2.33, R^2 = .254, *adjusted* R^2 = .145, *p (model)* = .050, *adjusted p* = .225

**Predictor**	***B***	***SE***	***t***	***p (predictor)***
**Quality of life**	11.68	7.50	1.56	.127
**Physical health**	1.70	7.25	0.23	.816
**Functioning**	-14.37	7.29	-2.02	.050
**Cognition**	1.44	7.12	0.20	.841
**Clinical symptoms**	11.39	5.96	1.91	.063
**Brain function**	-1.74	5.73	-0.30	.763

Model 9: Satisfaction with exercise training

N = 41, *F* (1, 39) = 0.52, R^2 = .013, *adjusted* R^2 = -.012, *p (model)* = .477, *adjusted p* = .895

**Predictor**	***B***	***SE***	***t***	***p (predictor)***
**Satisfaction with exercise training**	0.70	0.97	0.72	.477

Multiple linear regression model predicting PWC

Model 10: Personality traits as predictors of PWC

N = 65, *F* (5, 59) = 0.38, R^2 = .032, *adjusted* R^2 = -.051, *p (model)* = .858

**Predictor**	***B***	***SE***	***t***	***p (predictor)***
**Extraversion**	-0.02	0.09	-0.23	.820
**Agreeableness**	-0.06	0.09	-0.74	.463
**Conscientiousness**	0.08	0.10	0.83	.410
**Neuroticism**	-0.06	0.08	-0.70	.486
**Openness**	-0.02	0.07	-0.24	.815

Note. *N* denotes the effective sample size for each model. *B* represents the unstandardized regression coefficient, *SE* denotes the standard error, and t denotes the t-test statistic for individual regression coefficients. *F* denotes the overall model test statistic. R^2 *and adjusted* R^2 represent the coefficient of determination and adjusted coefficient of determination, respectively. The *p* (predictor) refers to the significance of individual regression coefficients, *p* (model) refers to the significance of the overall regression model. The adjusted *p*-values were calculated using the Benjamini-Hochberg procedure for all regression models. Abbreviations: EMI-2, Exercise Motivation Inventory-2; GAF, Global Assessment of Functioning; IE-4, Intrinsic Exercise Motivation Scale; PANSS, Positive and Negative Syndrome Scale; PWC, Physical Working Capacity; SSA, Self-efficacy towards physical exercise.

Additional Sensitivity Analyses

Sensitivity analyses were performed by incorporating study membership (BT vs. OT) as a covariate in all regression models. Models that included variables assessed solely within the BrainTrain cohort were excluded from these analyses, as adjusting for the study origin was not feasible. The results were largely consistent with those of the main analyses. Fitness remained the only model that achieved statistical significance at the uncorrected level (F (2,73) = 4.36, R^2 = .107, *adjusted* R^2 = .082, p = .016). Study membership did not show a significant association with training adherence across models. A similar sensitivity analysis of the exploratory fitness outcome produced comparable results, confirming the robustness of the findings.

Table S4. Regression models adjusted for study origin (BT vs. OT)

Model	*N*	*F*	R^2	*adjusted* R^2	*p* (model)	Study B (*B*)	*p* (study)
**Internal Motivation**	**62**	(4, 57) = 0.30	.021	-.048	.876	-7.32	.404
**Personality traits**	**65**	(6, 58) = 0.90	.085	-.010	.504	-7.34	.417
**Fitness**	**76**	(2, 73) = 4.36	.107	.082	.016	-9.18	.228
**Symptom severity (PANSS subscales)**	**76**	(4, 71) = 0.55	.030	-.025	.699	-7.42	.359
**Symptom severity (PANSS total score)**	**76**	(2, 73) = 0.69	.019	-.008	.506	-7.54	.343
**Functioning in daily life**	**75**	(2, 72) = 0.51	.014	-.014	.606	-7.63	.344
**Personality traits (PWC)**	**65**	(6, 58) = 0.73	.070	-.026	.629	0.25	.127

Note. Models including variables assessed exclusively in the BrainTrain cohort are not presented. Each row represents a separate multiple linear regression model. *N* refers to the effective sample size for each model. *F* denotes the overall model test statistic. R^2 *and adjusted* R^2 represent the coefficient of determination and adjusted coefficient of determination, respectively. The study variable comparing OT and BT was dummy coded with BT serving as the reference group. The regression coefficient (*B*) indicates the difference between the OT and BT. The term *p* (model) denotes the significance of the overall model, whereas *p* (study) refers to the significance of the study covariate. Abbreviations: PANSS, Positive and Negative Syndrome Scale; PWC, Physical Working Capacity.

Supplementary sensitivity analyses used a different adherence measure that accounted for all missed training sessions, excluding absences due to vacation. The results were largely consistent with the primary analyses. Fitness was the sole model to achieve uncorrected statistical significance (F (1, 74) = 7.68, R^2 = .094, *adjusted* R^2 = .082, p= .007). The results exhibited only minor changes, suggesting that the findings were resilient to the operationalization of training adherence.

Table S5. Sensitivity analyses assessing robustness of training adherence models using an alternative adherence definition

**Model**	***N***	***F***	***R²***	***adjusted R²***	***p***
**Internal motivation**	**62**	(3, 58) = 0.11	.006	-.046	.954
**Personality traits**	**65**	(5, 59) = 0.75	.060	-.020	.590
**Fitness**	**76**	(1, 74) = 7.68	.094	.082	.007
**Previous experiences and emotional support**	**49**	(3, 45) = 0.07	.005	-.062	.976
**Symptom severity (PANSS subscales)**	**76**	(3, 72) = 0.57	.023	-.018	.639
**Symptom severity (PANSS total score)**	**76**	(1, 74) = 0.66	.009	-.005	.419
**Functioning in daily life**	**75**	(1, 73) = 0.06	.001	-.013	.811
**Affective forecasting**	**48**	(6, 41) = 1.58	.188	.069	.177
**Satisfaction with exercise training**	**41**	(1, 39) = 0.20	.005	-.020	.658

Note. *N* refers to the effective sample size for each model. *F* denotes the overall model test statistics. *R^2 and adjusted R^2* represent the coefficient of determination and adjusted coefficient of determination, respectively. *p* refers to the overall model significance. Abbreviation: PANSS, Positive and Negative Syndrome Scale.

References

- Fuchs, R., and Schwarzer, R. (1994). Selbstwirksamkeit zur sportlichen Aktivität: Reliabilität
  und Validität eines neuen Meßinstruments [self-efficacy towards physical exercise: reliability and validity of a new instrument]. *Z. Differ. Diagn. Psychol*. 15, 141–154. Available at: https://www.sport.uni-freiburg.de/de/institut/psychologie/messinstrumente/selbstwirksamkeitsportlicheaktivi.pdf (Accessed: May 9, 2026)

- Jones, S.H., Thornicroft, G., Coffey, M., Dunn, G. (1995). A brief mental health outcome scale – reliability and validity of the Global Assessment of Functioning (GAF). *British Journal of Psychiatrie*, 166, 654-659. doi: 10.1192/bjp.166.5.654

- Kay, S. R., Fiszbein, A., and Opler, L. A. (1987). The Positive and Negative Syndrome Scale (PANSS) for Schizophrenia. *Schizophr. Bull.* 13, 261–276. doi: 10.1093/schbul/13.2.261

- Kim, H.-R., Woo, S.-H., Seo, J.-P., So, W.-Y. and Bae, J.-S. (2024). Satisfaction with the exercise program and successful aging among older adults who exercise regularly: the multiple mediation of physical self-efficacy and exercise adherence. *Healthcare*, 12: 2054. doi: 10.3390/healthcare12202054

- Kovaleva, A., Beierlein C., Kemper C.J., Rammstedt, B. (2012). Eine Kurzskala zur Messung von Kontrollüberzeugung: Die Skala Internale-Externale-Kontrollüberzeugung-4 (IE-4) [A short scale for measuring control beliefs: The Internal-External Control Beliefs Scale-4 (IE-4)]. GESIS Working Papers No. 2012-19. Available at: https://www.gesis.org/fileadmin/upload/forschung/publikationen/gesis_reihen/gesis_arbeitsberichte/WorkingPapers_2012-19.pdf (Accessed March 15, 2026)

- Markland D, Hardy L. The Exercise Motivations Inventory: Preliminary Development and Validity of a Measure of Individuals' Reasons for Participation in Regular Physical Exercise. *Personality and Individual Differences* (1993) 15(3):289-96. doi: 10.1016/0191-8869(93)90219-S

- Markland, D., and Ingledew, D. K. (1997). The measurement of exercise motives: Factorial validity and invariance across gender of a revised Exercise Motivations Inventory. *Br. J. Health Psychol.* 2, 361–376. doi: 10.1111/j.2044-8287.1997.tb00549.x

- Rammstedt, B., Kemper, C. J., Klein, M. C., Beierlein, C., Kovaleva, A. (2013). A Short Scale for Assessing the Big Five Dimensions of Personality: 10 Item Big Five Inventory (BFI-10). *Methods, Data, Analyses*,7(2), 233-249. doi: 10.12758/mda.2013.013

- Wilson, T. D. and Gilbert, D. T. (2003). Affective forecasting. In: M.P. Zanna, (Ed.), Advances in experimental social psychology, Vol. 35, 345–411. Elsevier Academic Press. doi: 10.1016/S0065-2601(03)01006-2
